# Rosemary Extract Reduces Odor in Cats Through Nitrogen and Sulfur Metabolism by Gut Microbiota–Host Co-Modulation

**DOI:** 10.3390/ani15142101

**Published:** 2025-07-16

**Authors:** Ziming Huang, Miao Li, Zhiqin He, Xiliang Yan, Yinbao Wu, Peiqiang Mu, Jun Jiang, Xu Wang, Yan Wang

**Affiliations:** 1 State Key Laboratory of Swine and Poultry Breeding Industry, College of Animal Science, South China Agricultural University, Guangzhou 510642, China; h2473552637@stu.scau.edu.cn (Z.H.); 15521277019@163.com (M.L.); 15360428153@163.com (Z.H.); yanxiliang1991@163.com (X.Y.); wuyinbao@scau.edu.cn (Y.W.); 2 Guangdong Provincial Key Lab of Agro-Animal Genomics and Molecular Breeding, South China Agricultural University, Guangzhou 510642, China; 3 National Engineering Research Center for Breeding Swine Industry, South China Agricultural University, Guangzhou 510642, China; 4 College of Life Science, South China Agricultural University, Guangzhou 510642, China; mpeiqi-ang@scau.edu.cn (P.M.); jiangjun@scau.edu.cn (J.J.); 5 Institute of Quality Standard and Monitoring Technology for Agro-Products of Guangdong Academy of Agricultural Sciences, Guangzhou 510642, China; wangxuguangzhou@126.com

**Keywords:** rosemary extracts, pet cats, ammonia (NH_3_), hydrogen sulfide (H_2_S), odor reduction

## Abstract

Odors from pet cats can negatively affect the quality of life of cat owners. The diverse bioactive compounds in plant extracts make them a promising candidate for effective odor reduction. This study evaluated twelve plant extracts for deodorizing efficacy via in vitro fermentation tests. Rosemary extract and licorice extract exhibited better deodorizing effects, with fractions of rosemary extract below 100 Da demonstrating the most effective deodorizing performance. Based on these findings, subsequent feeding trials were conducted using rosemary extract and its fractions below 100 Da. In the feeding trial, adult British Shorthair cats were divided into three groups (Control Check, RE, and RE100) and housed in a controlled-environment respiration chamber for 30 days. Measurements included odor emissions, fecal and blood physicochemical parameters, immune parameters, microbiota composition based on 16S rRNA sequencing, and metabolome analysis. The results of the feeding trial indicated that rosemary extract significantly reduced ammonia and hydrogen sulfide emissions (46.84%, 41.64%), while fractions below 100 Da of rosemary extract achieved even greater reductions (55.62%, 53.87%). Rosemary extract regulated the intestinal microbial community, significantly increasing the relative abundance of the intestinal probiotic *Bifidobacterium* (*p* < 0.05) and reducing the population of sulfate-reducing bacteria (*p* < 0.05). It also significantly reduced urease and uricase activities (*p* < 0.05) to reduce ammonia production and inhibited the degradation of sulfur-containing proteins and sulfate reduction to reduce hydrogen sulfide emissions. Furthermore, rosemary extract significantly enhanced the immune function of British Shorthair cats (*p* < 0.05). This study suggests that rosemary extract, particularly its fractions below 100 Da, is a highly promising pet deodorizer.

## Simple Summary

Odorous emissions from pet cats are an important contributor to the quality of life of cat owners. Plant extracts rich in bioactive compounds offer a potentially effective solution to this problem. This study evaluated twelve plant extracts via in vitro fermentation, identifying rosemary and licorice as the most effective. Further analysis revealed that rosemary fractions below 100 Da (Dalton) had the best deodorizing effect. Building on these findings, feeding trials were conducted to evaluate the practical effectiveness of rosemary extract in reducing odor emissions and to explore its underlying mechanisms. In feeding trials with British Shorthair cats, rosemary extract, particularly its fractions below 100 Da, significantly reduced ammonia and hydrogen sulfide emissions. It reduced odor emissions by decreasing urease and uricase activity, inhibiting sulfur-containing protein degradation and sulfate reduction, while increasing the relative abundance of the intestinal probiotic (*Bifidobacterium*) and enhancing immune function. These results suggest that rosemary extract, particularly its fractions below 100 Da, is a promising natural pet deodorizer.

## 1. Introduction

In recent years, the number of pet cats has steadily increased [1]. As companion animals, they play a important role in supporting human psychosocial well-being [2]. However, odor issues associated with cat ownership also pose challenges for people [3]. Odor emissions not only degrade indoor air quality and negatively impact the living environment for both owners and pets, but also pose health risks by increasing the risk of respiratory and cardiovascular conditions. These include signs such as respiratory distress, appetite loss, nausea, and headaches [4,5,6]. Therefore, research on deodorization techniques for pet cats has the dual practical value of improving the quality of human–pet cohabitation and mitigating public health risks.

Odorous gases emitted by pet cats exhibit complex and diverse chemical compositions, primarily consisting of nitrogenous, sulfurous, and aliphatic compounds [7,8]. Among these, ammonia (NH_3_) and hydrogen sulfide (H_2_S) are the primary contributors to odor [9], making their mitigation highly important in practice. Microbial catabolism of uric acid in the intestine is the primary biosynthetic pathway for NH_3_ [10], with enzymes such as uricase and urease playing key roles. Bacteria such as *Escherichia coli* and *Candida utilis* exhibit high uricase activity [11], facilitating the efficient conversion of uric acid into urea. Urea is subsequently hydrolyzed into NH_3_ by urease-producing bacteria, including *Clostridium*, *Proteus*, and *Klebsiella* [12]. Additionally, amino acid deamination serves as a secondary source of NH_3_ [13], with its metabolic activity positively correlated with dietary protein intake [14]. This suggests that enhancing protein digestibility could help reduce the occurrence of deamination reactions. H_2_S is mainly produced through microbial degradation of sulfur-containing amino acids and the activity of sulfate-reducing bacteria on inorganic sulfur [15,16]. Major contributors include *Vibrio desulfuricans*, *Verrucomicrobium*, *Megacoccus*, and *Enterobacteriaceae* [17,18,19]. Based on these mechanisms, odor emission can be effectively mitigated by inhibiting odor-related enzymatic activities, enhancing protein and amino acid digestibility, and inhibiting the growth of odor-producing microorganisms.

Plant extracts contain a diverse array of bioactive compounds, including polysaccharides, polyphenols, saponins, alkaloids, terpenes, and volatile oils [20,21,22]. Acting synergistically within the animal body, these compounds exhibit a wide range of effects, including broad-spectrum antibacterial [23], antioxidant [24], immunomodulatory [25], intestinal regulatory activities [26]. These multifunctional properties endow plant extracts with strong potential for reducing odor emissions. A variety of plant extracts, including *Yucca*, garlic, tea, cinnamon, and *Astragalus* [27,28,29,30,31], have demonstrated efficacy in odor mitigation. Due to their natural safety, renewability, and ease of application, plant extracts are increasingly utilized in the pet care industry. However, existing studies on the deodorization of pet cats by plant extracts are still mainly focused on *Yucca extracts* [32,33]. Moreover, comparative evaluations of different plant extracts remain limited, and their underlying deodorization mechanisms are yet to be fully elucidated. These gaps should be taken into account and addressed in future practical applications.

In this study, we evaluated the odor-reducing efficacy of twelve plant extracts and their molecular weight fractions via in vitro fermentation, followed by validation through a feeding trial. Furthermore, we explored the underlying mechanisms of odor reduction by analyzing the physicochemical properties of fresh feces and serum, as well as the gut microbial community composition via high-throughput sequencing. This study broadens the application scope of plant extracts in the pet industry and provides theoretical support and scientific guidance for reducing odor emissions from pet cats.

## 2. Materials and Methods

### 2.1. Materials

The twelve plant extracts used in this study included garlic extract (GE), *Salvia officinalis extract* (SOE), green tea extract (GTE), honeysuckle extract (HE), orange peel extract (OPE), thyme extract (TE), *Yucca extract* (YE), cinnamon extract (CE), *Astragalus extract* (AE), black tea extract (BTE), rosemary extract (RE), and licorice extract (LE). All extracts were purchased from Nanjing Herbal Source Biotechnology Co. (Nanjing, China). RE and LE were further fractionated by molecular weight via ultrafiltration, following the procedure described by Neagu et al. [34]. RE was divided into four fractions: RE1 (<100 Da), RE2 (100–500 Da), RE3 (500–1000 Da), and RE4 (>1000 Da). Similarly, LE was separated into four fractions: LE1 (<3500 Da), LE2 (3500–7000 Da), LE3 (7000–14,000 Da), and LE4 (>14,000 Da). All cat food used was complete cat food produced by Guangzhou Shengnuo Trading Co. (Guangzhou, China).

### 2.2. In Vitro Fermentation Test

Fresh fecal samples were collected from six healthy adult British Shorthair cats for use in in vitro fermentation tests. The in vitro fermentation system was established following the methodology described by Bosch et al. [35]. Within 15 min of defecation, feces were transferred to 50 mL centrifuge tubes pre-filled with CO_2_. The tubes were then immediately flushed with CO_2_ to maintain anaerobic conditions. Any cat litter adhering to the feces was manually removed. The samples were then weighed and diluted at a ratio of 1:9 (weight/volume) with sterile saline solution (9 g/L NaCl) at 39 °C. The mixture was stirred under anaerobic conditions and filtered through four layers of sterile gauze to obtain the filtrate. This filtrate was incubated at 39 °C in a water bath with continuous CO_2_ infusion, serving as the microbial inoculum for fermentation.

The inoculum solution was prepared following the method outlined by Williams et al. [36]. It was then mixed with the previously prepared filtrate at a volume ratio of 84:5 to form the in vitro fermentation mixture. As the fermentation substrate, 500 mg of feed was accurately weighed and slowly transferred to the bottom of a syringe using a paper strip. Twelve kinds of plant extracts (0.1%) were subsequently added and thoroughly mixed. The control check group (CK) received no plant extracts but underwent the same procedure. Each experimental group was established with five replicates. Each syringe was filled with 30 mL of the fermentation mixture, and syringe pistons were evenly coated with petroleum jelly and carefully inserted to prevent substrate expulsion during fermentation. Rubber stoppers were used to maintain anaerobic conditions within the syringes, which were then incubated in a thermostatic shaker at 39 °C and 90 rpm for 24 h. After incubation, an ice bath was used to terminate fermentation. The volume of gas produced in each syringe was recorded, and the gas was immediately transferred into sulfuric acid and hydrogen sulfide absorption solutions to collect NH_3_ and H_2_S, respectively.

### 2.3. Animal Experimental Design

Nine healthy adult British Shorthair cats were used in the feeding trial. Before the trial began, all cats were housed under standardized conditions at the Laboratory Animal Center of South China Agricultural University, where they were fed the same type and amount of feed daily and provided with ample clean drinking water. Following a completely randomized design, cats were randomly assigned to three groups: the control check group (CK group, n = 3), the group supplemented with RE (RE group, n = 3), and the group supplemented with RE1 (RE100 group, n = 3). Each treatment group included three cats. The feeding trial was conducted using respiratory metabolism chambers, with one cat housed per chamber. Cats were fed a fixed amount of food at 9:00 and 17:00 daily and provided with unlimited access to clean drinking water. Each cat was weighed at the beginning and end of the trial. During the trial, the CK group received commercial cat food; the RE group received commercial cat food supplemented with 0.1% RE; and the RE100 group received commercial cat food supplemented with 0.1% RE1.

The 4-week feeding trial was divided into a three-week pre-feeding period and a one-week respiratory test. During the respiratory test, an outer plexiglass cover was placed over the metabolic chambers. The first four days allowed the cats to adapt to their new environment, followed by a three-day formal respiratory measurement period. Throughout the testing phase, each respiratory metabolism chamber was treated as a unit for daily recording of food intake. On the morning of the fifth day of the gas collection test, fresh and uncontaminated feces were collected from each cat, placed into 5 mL cryotubes, immediately stored in liquid nitrogen, and subsequently transferred to a −80 °C freezer for storage. On the morning following the completion of the gas collection test, fasting blood samples (5 mL) were collected from the forelimb vein of each cat. The blood samples were allowed to sit at room temperature for 30 min, then centrifuged at 3500 rpm for 15 min at 4 °C. The resulting serum was collected and stored at −80 °C for further analysis.

### 2.4. Respiratory Test

Respiratory metabolism chambers (Patent Application No. 201610091580.9), designed by our laboratory, were modified for use in the feeding trial to measure NH_3_ and H_2_S emissions during the feeding process in British Shorthair cats. The structure of these chambers is illustrated in Figure S1.

The outer plexiglass cover was placed over the bottom plate, and the gas absorption bottle, gas pump, flow meter, and other components were connected to ensure the airtightness of the chamber. The gas pump was then started, and the gas flow meter was used to regulate the exhaust flow and maintain fresh air circulation. After a four-day acclimatization period, formal gas collection was initiated. During the test, the sulfuric acid and hydrogen sulfide absorption solutions were replaced every two hours-twelve times per day-for three consecutive days. During each replacement, the air pump was temporarily turned off and then restarted to restore airflow. A 10 mL sample was collected from each replaced sulfuric acid and hydrogen sulfide absorption solution into centrifuge tubes for subsequent NH_3_ and H_2_S analysis. Temperature and humidity inside and outside the test room, as well as within each respiratory metabolism chamber, were recorded daily. Researchers remained on duty 24 h a day to monitor the cats’ health status and promptly clean feces.

### 2.5. Measurement of Physical and Chemical Indexes of Odor Absorbing Liquid and Fresh Feces

The concentration of NH_3_ collected in the sulfuric acid absorption solution, as well as ammonium nitrogen, nitrate nitrogen, uric acid, urea, uricase activity, urease activity, pH, and electrical conductivity in fresh feces, were determined according to the methodology described by Wang et al. [37].

Similarly, the concentration of H_2_S collected in the hydrogen sulfide absorption solution, the population of sulfate-reducing bacteria, sulfate ion concentration, and the expression of methionine γ-cleavage enzyme mRNA in fresh feces were assessed following the method established by Deng et al. [38].

### 2.6. Measurement of Blood Physical and Chemical Indices

Serum samples were used to analyze blood biochemical parameters. Levels of immunoglobulin A (IgA), immunoglobulin M (IgM), immunoglobulin G (IgG), interleukin-6 (IL-6), and interleukin-10 (IL-10) were measured using commercial ELISA kits (MLbio Co., Ltd., Shanghai, China). Concentrations of blood ammonia, uric acid, urea, and xanthine oxidase were measured using commercial assay kits (Nanjing Jiancheng Biotechnology Research Institute, Nanjing, China; kit numbers: C012-1-1, C013-1-1, A086-1-1, and A002-1-1). All assays were performed according to the manufacturer’s instructions.

### 2.7. Fecal Microbiota Analysis

Genomic DNA was extracted from fecal samples using the Bacterial DNA Kit (OMEGA Biotek, Norcross, GA, USA). For each cat, two replicate samples were collected, resulting in six replicates per group. The 16S rRNA gene, including the V3 and V4 hypervariable regions, was amplified by PCR using bacterial-specific primers 515F (5′-GTGYCAGCMGCCGCGGTAA-3′) and 806R (5′-GGACTACNVGGGTWTCTAAT-3′). The PCR thermal cycling conditions were as follows: initial denaturation at 95 °C for 60 s (1 cycle), followed by 40 cycles of denaturation at 95 °C for 15 s, annealing at 56 °C for 15 s, and extension at 72 °C for 40 s, with a final extension at 72 °C for 10 min. DNA concentrations were quantified, and sequencing libraries were constructed using the TruSeq^®^ DNA PCR-Free Sample Preparation Kit (Illumina, CA, USA). Sequencing was performed on the NovaSeq 6000 platform (Novogene, Beijing, China). Raw paired-end reads were merged using FLASH and demultiplexed based on unique sample barcodes. High-quality reads were filtered and clustered into operational taxonomic units (OTUs) at 97% sequence similarity using UCLUST (Robert C. Edgar, Tiburon, CA, USA).

Sequencing data were analyzed to evaluate both alpha and beta diversity. Alpha diversity was evaluated using Chao1 index and Shannon index to measure species richness and evenness within groups. Beta diversity was evaluated using principal coordinate analysis (PCoA) based on Bray–Curtis similarity to compare differences in microbial community composition between groups.

### 2.8. Untargeted Metabolomics Analysis

Untargeted metabolomics analysis was conducted on the plant extracts. Approximately 50 ± 1 mg of each sample was transferred to a 2 mL microcentrifuge tube, and 1000 μL of pre-cooled extraction solvent (n-hexane containing an internal standard) was added. The mixture was vortexed for 30 s, followed by the addition of a steel bead and homogenized at 35 Hz for 4 min. Ultrasonic treatment was then performed in an ice-water bath for 5 min, repeated three times. After centrifugation at 4 °C for 15 min at 12,000 rpm (RCF = 13,800× *g*, R = 8.6 cm), 150 μL of the supernatant was transferred to a clean tube for analysis using a gas chromatograph coupled with time-of-flight mass spectrometer (GC-TOF-MS, LECO Corporation, St. Joseph, MI, USA). Analysis was performed on a SHIMADZU GC-2020 system (J&W Scientific, Folsom, CA, USA) equipped with a DB-5MS capillary column. A 1 μL aliquot was injected in splitless mode. Helium was used as the carrier gas, with a purge flow of 3 mL/min and a column flow rate of 1 mL/min. The initial oven temperature was held at 50 °C for 1 min, then increased to 310 °C at a rate of 8 °C/min, and maintained at 310 °C for 11.5 min. The injection port, transfer line, and ion source temperatures were set at 280 °C, 280 °C, and 200 °C, respectively. Electron impact ionization was performed at −70 eV. Mass spectrometry data were acquired in full-scan mode over an *m*/*z* range of 50–500 at 12.5 spectra per second, following a solvent delay of 7.2 min.

### 2.9. Statistical Analysis

Statistical analysis and initial data categorization were performed using Excel 2010 (Microsoft, Redmond, WA, USA) and SPSS 22.0 (IBM, Armonk, NY, USA). One-way ANOVA followed by Duncan’s multiple range test was conducted to assess differences among groups. Results are presented as mean ± standard error of the mean (SEM), with statistical significance set at *p* ≤ 0.05. Bar charts, line graphs, and box plots were generated using GraphPad Prism 9.5 (GraphPad Software, San Diego, CA, USA) for data visualization. For microbial community analysis, 16S rRNA gene sequencing data were processed using QIIME version 1.9.1 (QIIME Development Team, Boulder, CO, USA). Spearman correlation analyses between NH_3_ and H_2_S concentrations and bacterial community composition were performed using OriginPro 2025 (OriginLab, Northampton, MA, USA), and the results were visualized as correlation heatmaps. Raw metabolomics data were processed using Chroma TOF 4.3X software (LECO Corporation, St. Joseph, MI, USA) in comjunction with the NIST database.

## 3. Results

### 3.1. Odor Reduction of In Vitro Fermentation

**Figure 1:**
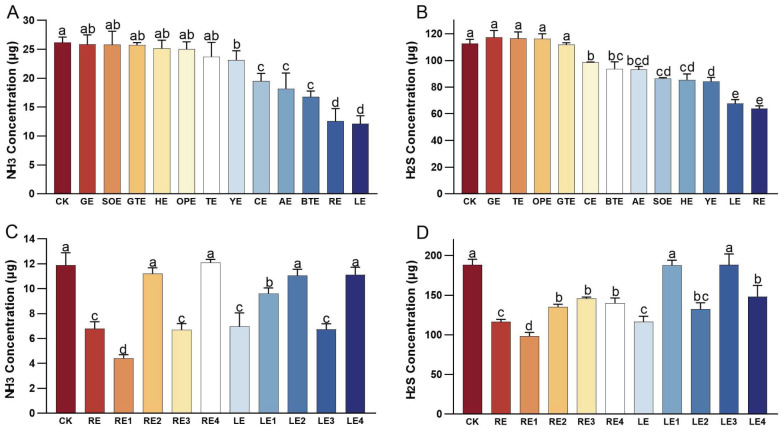
Emissions of NH_3_ and H_2_S from different treatment groups in the in vitro fermentation test. (**A**) Effect of various plant extracts on NH_3_ production in vitro. (**B**) Effect of various plant extracts on H_2_S production in vitro. (**C**) Effect of plant extract fractions with different molecular weight on NH_3_ production in vitro. (**D**) Effect of plant extract fractions with different molecular weight on H_2_S production in vitro. Error bars represent the standard error of the mean (n = 5). Different letters above the bars indicate statistically significant differences between treatments (ANOVA and Duncan test, *p* < 0.05).

The initial in vitro fermentation experiment demonstrated varying levels of odor mitigation among different plant extracts (Figure 1A,B). Compared with the CK group, NH_3_ production was significantly decreased in the YE, CE, AE, BTE, RE, and LE groups (*p* < 0.05). Among these, the RE and LE groups exhibited the most pronounced reductions in NH_3_ production, with decreases of 52.02% and 53.62%, respectively. The LE group showed the greatest reduction overall reduction. Similarly, H_2_S production was significantly lower in the CE, BTE, AE, SOE, HE, YE, LE, and RE groups compared to the CK group (*p* ≤ 0.05). The LE and RE groups again demonstrated the most substantial reductions, with emission rates decreased by 39.86% and 43.34%, respectively. The RE group had the best effect on reducing H_2_S emissions. Based on the efficacy of odor reduction, the RE and LE groups were selected for further investigation of the effects of different molecular weight fractions on odor mitigation.

The second in vitro fermentation experiment demonstrated varying levels of odor mitigation among different molecular weight fractions of the plant extracts (Figure 1C,D). NH_3_ production was significantly reduced in the RE, RE1, RE3, LE, LE1, and LE3 groups compared to the CK group (*p* ≤ 0.05). The RE1 group exhibited the most pronounced reduction, with a 63.02% decrease in NH_3_ production. Similarly, H_2_S production was significantly lower (*p* ≤ 0.05) in the RE, RE1, RE2, RE3, RE4, LE, LE2, and LE4 groups than in the CK group. The RE1 group again demonstrated the greatest reduction, with a 47.84% decrease in H_2_S production. Given its superior performance in odor abatement, the molecular weight of the rosemary extracts that were below 100 Da (RE1, designated as RE100 in the feeding trial) was selected for the feeding trial.

### 3.2. Odor Reduction of Cat Breeding Experiments

**Figure 2:**
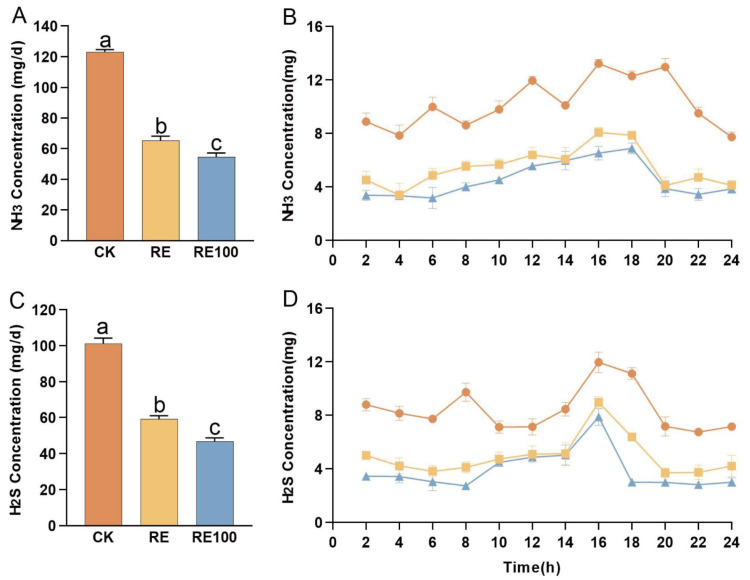
Emissions of NH_3_ and H_2_S from different treatment groups in the feeding test. (**A**) NH_3_ emissions from different treatment groups during the feeding test. (**B**) Temporal trends in NH_3_ emissions among different treatment groups during the feeding test. (**C**) H_2_S emissions from different treatment groups during the feeding test. (**D**) Temporal trends in H_2_S emissions among different treatment groups during the feeding test. Error bars represent the standard error of the mean (n = 3). Different letters above the bars indicate statistically significant differences between treatments (ANOVA and Duncan test, *p* < 0.05).

ANOVA results indicated that both the RE and RE100 groups significantly reduced the NH_3_ production in British shorthair cats relative to the CK group, with emission reductions of 46.84% and 55.62%, respectively (*p* < 0.05; Figure 2A). Similarly, H_2_S production was significantly lower in the RE and RE100 groups, with respective reductions of 41.64% and 53.87% compared to the CK group (*p* < 0.05; Figure 2C). Additionally, we observed that both NH_3_ and H_2_S abatement were significantly better in RE100 than in the RE group (*p* < 0.05). The emission trends for NH_3_ and H_2_S are presented in Figure 2B,D, respectively. The emissions levels of both NH_3_ and H_2_S remained relatively stable throughout the observation period. Notably, the reductions in NH_3_ and H_2_S emissions were consistent across all time points after plant extracts supplementation, indicating a sustained and stable odor-mitigation effect in both the RE and RE100 groups.

### 3.3. Effects on Growth Performance and Serum Immune Parameters

The experimental results showed that neither the RE nor the RE100 group significantly affected the initial body weight, final body weight, average daily feed intake, or average daily gain in British Shorthair cats compared to the CK group (*p* > 0.05; Table S1). This lack of significance may be due to the relatively short feeding duration or the supplementation concentration not reaching the growth-promoting threshold. Additionally, the RE group significantly increased the serum levels of IgA and reduced the serum levels of IL-6 in British shorthair cats (*p* < 0.05), while the RE100 group significantly increased the serum levels of IgA, IgG, and IL-10, and significantly reduced the levels of IL-6 (*p* < 0.05; Table 1). These findings suggest that rosemary extract may enhance the immune function in British shorthair cats, and that fractions with molecular weights below 100 Da are more effective.

### 3.4. Effects on Fresh Feces and Serum Physicochemical Indicators

**Figure 3:**
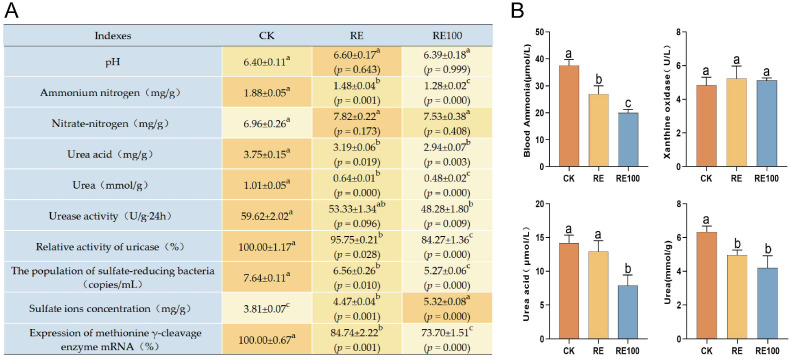
Physicochemical indicators of fresh feces and serum in different groups. (**A**) Physicochemical indicators of fresh feces across different groups. The orange background represents the average value, with darker shades indicating higher values. (**B**) Serum physicochemical indicators across different groups. Error bars represent the standard error of the mean (n = 3). Different letters above the bars indicate statistically significant differences between treatments (ANOVA and Duncan test, *p* < 0.05).

Pet cat odors are primarily emitted through feces [3]. This study showed that the RE and RE100 groups significantly reduced ammonium nitrogen, uric acid, urea, relative uricase activity, sulfate-reducing bacteria abundance, and methionine γ-cleavage enzyme mRNA expression, while increasing sulfate ion concentration in fresh feces of British shorthair cats (Figure 3A). Although nitrate nitrogen levels increased in both groups, the changes were not statistically significant (*p* > 0.05). Furthermore, the RE100 group significantly decreased urease activity in fresh feces (*p* < 0.05).

Serum concentrations of uric acid, urea, and ammonia are important indicators of nitrogen metabolism [39]. Inhibition of xanthine oxidase, a key enzyme in uric acid synthesis, can reduce uric acid production and help regulates ammonia metabolism. In this study, compared with the CK group, both the RE and RE100 groups significantly reduced blood ammonia (37.53 ± 1.28 μmol/L, 26.92 ± 1.78 μmol/L, 19.94 ± 0.78 μmol/L, *p* < 0.05) and urea concentrations (6.33 ± 0.21 mmol/L, 4.96 ± 0.17 mmol/L, 4.20 ± 0.42 mmol/L, *p* < 0.05). Additionally, the RE100 group significantly reduced uric acid concentration (14.19 ± 0.67 μmol/L, 12.90 ± 0.93 μmol/L, 7.85 ± 0.92 μmol/L, *p* < 0.05) in the British shorthair cats (Figure 3B). Although both RE and RE100 groups also reduced xanthine oxidase activity, the differences were not statistically significant (5.24 ± 0.42 U/L, 5.11 ± 0.09 U/L, 4.83 ± 0.28 U/L, *p* > 0.05).

### 3.5. Effects on the Microbiome Community of Fresh Feces

**Figure 4:**
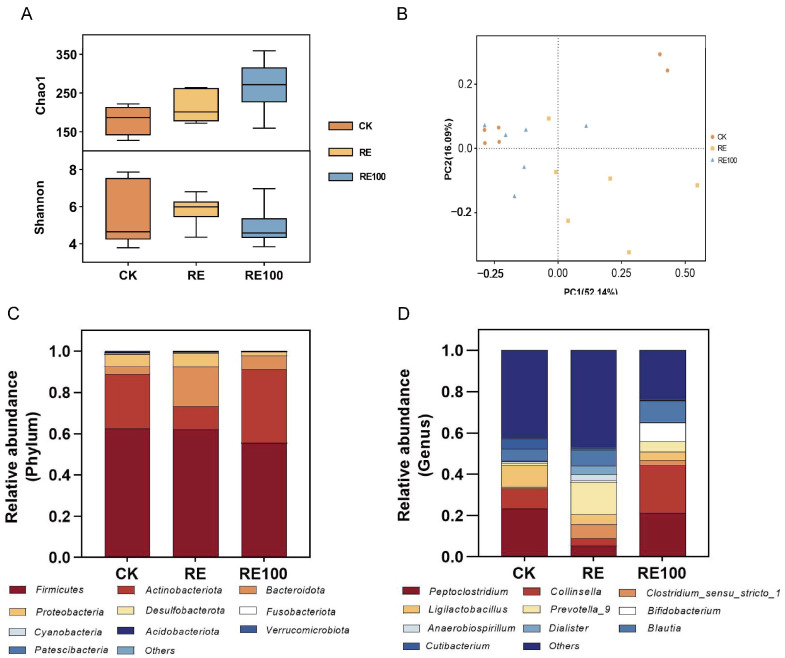
Structure and diversity of fresh fecal microbiota across different treatment groups. (**A**) Bacterial α-diversity. (**B**) Bacterial β-diversity. (**C**) Microbial community composition at the phylum level. (**D**) Microbial community composition at the genus level.

This study employed 16S rRNA sequencing to evaluate the abundance and structure of the microbiome under various treatments. The α-diversity of the microbiome in fresh feces is shown in Figure 4A. The Chao1 index values were as follows: CK (180.49 ± 19.56), RE (216.36 ± 19.62), and RE100 (268.49 ± 66.74). For the Shannon index, the values were CK (5.46 ± 0.71), RE (5.84 ± 0.33), and RE100 (4.88 ± 0.44). Intergroup differences in both the Chao1 and Shannon indices were not statistically significant (*p* > 0.05). PCoA based on weighted UniFrac distances was used to assess bacterial β-diversity (Figure 4B), and the results showed that microbiota composition differed among treatment groups.

The stacked bar charts showing microbiome composition at the phylum level are presented in Figure 4C, with Firmicutes, Actinobacteriota, Bacteroidota, and Proteobacteria identified as the dominant phyla. Compared with the CK group, the RE group exhibited a significantly lower relative abundance of Actinobacteriota and a higher abundance of Bacteroidota (*p* < 0.05). In contrast, the RE100 group exhibited a significantly higher relative abundance of Actinobacteriota than the RE group (*p* < 0.05; Figure S2). At the genus level, the top 10 genera across all groups were *Peptoclostridium*, *Collinsella*, *Clostridium_sensu_stricto_1*, *Ligilactobacillus*, *Prevotella_9*, *Bifidobacterium*, *Anaerobiospirillum*, *Dialister*, *Blautia*, *Cutibacterium* (Figure 4D). Relative to the CK group, the RE group significantly increased the relative abundance of *Prevotella_9*, *Parabacteroides*, *[Ruminococcus]_torques_group*, *Odoribacter* (*p* < 0.05), while decreasing that of *Catenisphaera* (*p* < 0.05). The RE100 group significantly increased *Bifidobacterium* and *Slackia* levels (*p* < 0.05), while significantly decreasing those of *Parabacteroides* and *[Ruminococcus]_torques_group* (*p* < 0.05). Compared to the RE group, the RE100 group further increased the relative abundance of *Peptoclostridium*, *Collinsella*, *Bifidobacterium*, and *Slackia* (*p* < 0.05), while reducing those of *Parabacteroides* and *[Ruminococcus]_torques_group* (*p* < 0.05; Figure S3). Despite these shifts in microbial composition, total bacterial counts remained statistically unchanged among the three groups (Table S2), suggesting that RE and RE100 selectively modulated specific functional microbes without affecting overall bacterial abundance.

### 3.6. Correlation Between Odor Concentrations and Bacterial Genera

**Figure 5:**
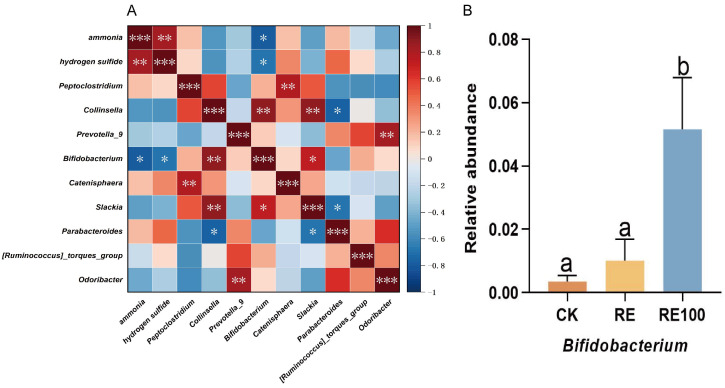
Correlation between odor concentrations and bacterial genera. (**A**) Heatmap of Spearman correlation analysis. Asterisks indicate statistically significant correlations (* 0.01 < *p* < 0.05, ** 0.001 < *p* < 0.01, *** *p* < 0.001). (**B**) Relative abundance of the odor-inhibiting bacteria. Error bars represent the standard error of the mean (n = 6). Different letters above the bars indicate statistically significant differences between treatments (ANOVA and Duncan test, *p* < 0.05).

Spearman correlation network analysis was conducted to investigate the associations between ammonia and hydrogen sulfide emissions and bacterial genera. Among the genera with significant changes in relative abundance, four were positively correlated, while five were negatively correlated with ammonia and hydrogen sulfide emissions (Figure 5A). Notably, a significant negative correlation was observed between *Bifidobacterium* and both gases. Although the relative abundance of *Bifidobacterium* was higher in the RE group than in the CK group, the difference was not statistically significant (*p* > 0.05). In contrast, the RE100 group exhibited a significantly higher relative abundance of *Bifidobacterium* compared to both the CK and RE groups (*p* < 0.05; Figure 5B).

### 3.7. Chemical Composition of Rosemary Extract

**Figure 6:**
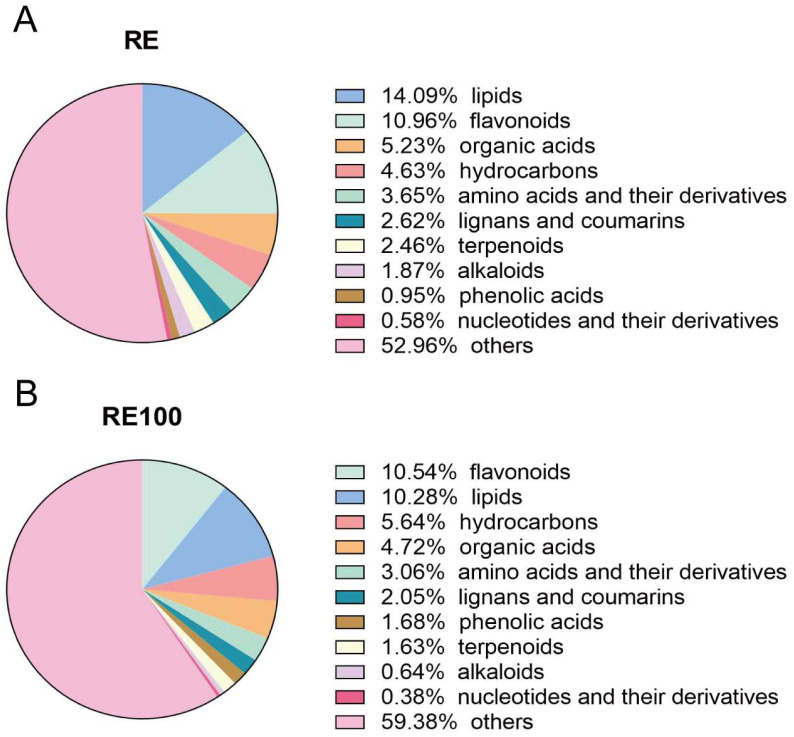
Classification of chemical compositions in rosemary extracts. (**A**) Chemical composition categories identified in the RE group. (**B**) Chemical composition categories identified in the RE100 group.

The RE and RE100 extracts used in the feeding trial were analyzed using GC-TOF-MS (LECO Corporation, St. Joseph, MI, USA) (Figure S4), and peak area normalization was applied for semi-quantitative analysis. The analytical workflow included peak extraction, baseline filtering and calibration, peak alignment, deconvolution, peak identification, integration, and spectral matching. In total, 148 chemical constituents were identified and classified into 11 categories (Figure 6), including 19 lipids, 12 flavonoids, 6 organic acids, 11 hydrocarbons, 12 amino acids and their derivatives, 5 lignans and coumarins, 10 alkaloids, 18 terpenoids, 4 phenolic acids, 4 nucleotides and their derivatives, and 47 unclassified compounds (Table S3).

## 4. Discussion

The odor reduction strategies for pet cats primarily focus on three approaches: source reduction, process control, and end-of-pipe treatment. Currently, the most widely adopted approach is end-of-pipe treatment, which involves masking odors using fragrances or adsorbent litter materials such as charcoal and bentonite clay [3,40]. However, this method provides only temporary relief and fails to address the root cause of odor generation. More effective solutions should target odor mitigation at the source by modifying dietary composition or incorporating functional feed additives. Incorporating plant extracts into pet diets has been shown to enhance metabolism, modulate gut microbiota, and reduce odor production. To this end, twelve candidate plant extracts were evaluated via in vitro fermentation to identify those with strong deodorizing potential. The results indicated that among the twelve extracts, RE and LE exhibited the most significant reductions in NH_3_ and H_2_S emissions. Further investigation identified RE1 (designated as RE100 in the feeding trial) as the most effective deodorizing fraction. Subsequently, an in vivo feeding trial was conducted to validate the deodorization effect of RE100. The feeding trial corroborated the in vitro fermentation findings, with NH_3_ and H_2_S emissions reduced by 46.84% and 41.64% in the RE group, respectively. In the RE100 group, the reductions were even more pronounced: 55.62% for NH_3_ and 53.87% for H_2_S. These results suggest that rosemary extract, particularly its fractions with molecular weights below 100 Da, holds strong potential for commercial deodorization applications in pet cats.

Rosemary extract appears to modulate both metabolic activity and immune function in British Shorthair cats. Blood ammonia, serum uric acid, and urea are key indicators to evaluate nitrogen metabolism efficiency in animals [39]. In this study, significant reductions in blood ammonia and urea levels were observed in the RE group, whereas the RE100 group exhibited marked decreases in blood ammonia, serum uric acid, and serum urea levels. These findings suggest that rosemary extract may enhance nitrogen utilization and reduce ammonia accumulation by improving the efficiency of protein metabolism. Additionally, the RE group showed a significant reduction in serum IL-6 levels, whereas the RE100 group exhibited significantly increased levels of IgA, IgG, and IL-10, along with reduced IL-6 levels. These findings indicate that rosemary extract may enhance immune responses through multi-targeted modulation, potentially mediated by the synergistic actions of its bioactive components, such as phenolic diterpenoids and flavonoids [41]. Notably, although previous studies have reported that rosemary extract can improve the growth performance of animals [42], no significant differences were observed among groups in terms of initial body weight, final weight, average daily feed intake, or average daily gain in the present study. This discrepancy may be due to the relatively short feeding duration or suboptimal dosage levels insufficient to elicit a growth-promoting effect.

The ability of rosemary extract to reduce NH_3_ production in British Shorthair cats may be attributed to its inhibitory effects on key ammonia-producing enzymes, such as uricase and urease. In the gastrointestinal tract, NH_3_ is primarily generated through microbial catabolism of uric acid, undigested proteins, and amino acids [43]. The activities of uricase and urease, the rate-limiting enzymes in these biochemical pathways, are positively correlated with NH_3_ production [44]. In this study, RE moderately reduced uricase relative activity and significantly inhibited urease activity, whereas RE100 significantly suppressed the activities of both enzymes. Uricase is an oxidoreductase whose activity is inhibited in a dose-dependent manner by various antioxidant compounds [45]. Chemical profiling of rosemary extract identified several antioxidant constituents, including flavonoids, alkaloids, phenolic acids, lignans, and coumarins. Among these, myricitrin (content: 9%), a flavonol glycoside with strong antioxidant properties [46], is hypothesized to be a major contributor to uricase inhibition by rosemary extract. Urease is a nickel-dependent enzyme whose activity can be inhibited by flavonoids and terpenoids through chelation of its metal-active site [47]. These findings support the conclusion that rosemary extract significantly inhibits the activities of uricase and urease, which may partially explain its efficacy in reducing NH_3_ emissions.

The modulatory effect of rosemary extract on H_2_S production in British Shorthair cats may involve both the inhibition of sulfur-containing protein degradation and sulfate-reducing bacterial proliferation. In carbohydrate deficiency, intestinal microbiota are stimulated to degrade sulfur-containing proteins such as methionine and cysteine via upregulation of methionine γ-cleavage enzymes synthesis, thereby producing H_2_S [48]. The mRNA expression of the methionine γ-cleavage enzyme serves as a marker of the metabolic activity involved in sulfur-containing protein degradation [49]. In this study, both the RE group and RE100 group significantly downregulated methionine γ-cleavage enzyme mRNA expression, suggesting a decreased reliance on sulfur-containing amino acid degradation for energy relative to the CK group. Additionally, specific amino acid residues such as cysteine and methionine may be oxidized by flavonoids present in the rosemary extract [50], thereby limiting the substrates available for H_2_S production. Sulfate-reducing bacteria utilize organic compounds as electron donors to convert sulfate into hydrogen sulfide [51]. Previous studies have demonstrated that rosemary effectively inhibits sulfate-reducing bacteria activity and mitigates the production of sulfur-containing gases [52]. In this study, both RE and RE100 groups significantly decreased the abundance of sulfate-reducing bacteria, leading in reduced H_2_S production via sulfate reduction and a corresponding increase in residual sulfate ions. Notably, the RE100 group exhibited a significantly greater reduction in sulfate-reducing bacteria abundance than the RE group, indicating a more substantial inhibition of sulfate-reducing activity. These two synergistic mechanisms may help explain the superior efficacy of rosemary extract in reducing H_2_S emissions compared to other plant extracts.

The composition of the intestinal microbiota in British Shorthair cats is closely linked to odor emissions. To explore this relationship, we analyzed the correlations between NH_3_ and H_2_S concentrations and bacterial genera, focusing on those with significant changes in relative abundance. Notably, *Bifidobacterium* exhibited a strong negative correlation with NH_3_ (r = −0.783) and H_2_S (r = −0.700) emissions. As a common intestinal probiotic, *Bifidobacterium* does not catabolize nitrogenous compounds but helps lower NH_3_ levels by inhibiting the urea cycle [53]. It also reduces H_2_S concentrations by limiting the proliferation of sulfate-reducing bacteria [54]. Additionally, *Bifidobacterium* promotes metabolic homeostasis within the gut microbiota, thereby contributing to overall odor mitigation [55]. In this study, the relative abundance of *Bifidobacterium* increased modestly in the RE group (not significant), whereas the RE100 group showed a statistically significant increase. These results suggest that rosemary extract may reduce odor emissions in British Shorthair cats by promoting the proliferation of *Bifidobacterium*.

A primary limitation of this study lies in its small sample size, which may limit the generalizability of the findings. Future studies involving larger sample sizes are warranted to validate and expand upon these findings.

## 5. Conclusions

In this study, twelve plant extracts were evaluated for odor reduction in British Shorthair cats, with rosemary and licorice extracts showing significant effects. Notably, the fractions of rosemary extract with molecular weight below 100 Da exhibited the greatest deodorizing efficacy. Rosemary extract can increase the relative abundance of intestinal probiotics *Bifidobacterium* and reduce the population of sulfate-reducing bacteria. It also decreased NH_3_ emissions by reducing the activities of uricase and urease, and reduced H_2_S emission by inhibiting the degradation of sulfur-containing proteins and sulfate reduction, and ultimately achieved the effect of odor emission reduction. However, the specific bioactive monomers responsible for these effects remain unclear. Therefore, further purification, separation, and validation of these active components are necessary. Additionally, rosemary extract was shown to enhance the immune function in British Shorthair cats. Future studies may also explore its potential benefits in improving coat quality, enhancing antioxidant capacity, and promoting growth and development, to fully maximize its functional properties. In conclusion, rosemary extract holds significant promise as a natural and effective solution for reducing odor emissions associated with pet cat ownership.

## Figures and Tables

**Table 1:** Effects of RE and RE100 on serum immune parameters in British shorthair cats.

	CK	RE	RE100
IgA (pg/mL)	14.13 ± 0.24 ^b^	19.41 ± 0.38 ^a^ (*p* = 0.003)	20.39 ± 1.05 ^a^ (*p* = 0.001)
IgM (pg/mL)	122.52 ± 0.42 ^a^	122.41 ± 1.14 ^a^ (*p* = 0.997)	121.50 ± 1.26 ^a^ (*p* = 0.768)
IgG (pg/mL)	43.75 ± 1.98 ^b^	45.97 ± 1.34 ^ab^ (*p* = 0.536)	50.16 ± 0.35 ^a^ (*p* = 0.040)
IL-6 (pg/mL)	71.05 ± 2.08 ^a^	57.09 ± 2.69 ^b^ (*p* = 0.023)	59.86 ± 3.11 ^b^ (*p* = 0.025)
IL-10 (pg/mL)	190.90 ± 6.96 ^b^	203.20 ± 5.01 ^b^ (*p* = 0.356)	261.01 ± 5.25 ^a^ (*p* = 0.000)

Values are expressed as mean ± SE (n = 3). Different superscripts letters (a, b) within the same row indicate statistically significant differences (*p* < 0.05).

## Data Availability

All the data generated or analyzed in this study are included in this paper. The 16S rRNA gene sequences in this study were deposited into the National Center for Biotechnology Information (NCBI) database (PRJNA1268125).

## Supplementary Materials

The following supporting information can be downloaded at: https://www.mdpi.com/article/10.3390/ani15142101/s1, Table S1. The growth performance of British Shorthair cats; Table S2. Abundance of total bacterial counts in fresh feces of different groups of British shorthair cats; Table S3. The chemical composition of RE and RE100. Figure S1. Schematic structure of the respiratory metabolism chamber; Figure S2. Differential analysis of phylum-level bacteria between the two groups; Figure S3. Differential analysis of genus-level bacteria between the two groups; Figure S4. A typical gas chromatogram of the chemical constituents of hexane extract.

## Abbreviations

The following abbreviations are used in this manuscript:

NH_3_	ammonia
H_2_S	hydrogen sulfide
Da	Dalton
CK	control check group
GE	garlic extract
SOE	*Salvia officinalis extract*
GTE	green tea extract
HE	honeysuckle extract
OPE	orange peel extract
TE	thyme extract
YE	*Yucca extract*
CE	cinnamon extract
AE	*Astragalus extract*
BTE	black tea extract
RE	rosemary extract
LE	licorice extract
RE1/RE100	fractions of rosemary extract (<100 Da)
RE2	fractions of rosemary extract (100–500 Da)
RE3	fractions of rosemary extract (500–1000 Da)
RE4	fractions of rosemary extract (>1000 Da)
LE1	fractions of licorice extract (<3500 Da)
LE2	fractions of licorice extract (3500–7000 Da)
LE3	fractions of licorice extract (7000–14,000 Da)
LE4	fractions of licorice extract (>14,000 Da)
IgA	Immunoglobulin A
IgM	Immunoglobulin M
IgG	Immunoglobulin G
IL-6	Interleukin-6
IL-10	Interleukin-10
OTUs	operational taxonomic units
PCoA	principal coordinate analysis
SEM	standard error of the mean
GC-TOF-MS	gas chromatograph coupled with a time-of-flight mass spectrometer
