# Supplementary material for: Rosemary Extract Reduces Odor in Cats Through Nitrogen and Sulfur Metabolism by Gut Microbiota–Host Co-Modulation
Source: Animals (Basel). 2025 Jul 16;15(14):2101. doi: 10.3390/ani15142101 (PMC12291628; doi:10.3390/ani15142101)
Supplement: Supplementary file 1 [file animals-15-02101-s001.zip › animals-3698521-supplementary.pdf]

animals

MDPI

Article

Rosemary Extract Reduces Odor in Cats Through Nitrogen and Sulfur Metabolism by Gut Microbiota–Host Co-Modulation

Ziming Huang [1], Miao Li [1], Zhiqin He [1], Xiliang Yan [1,2,3], Yinbao Wu [1,2,3], Peiqiang Mu [4], Jun Jiang [4], Xu Wang [5] and Yan Wang [1,2,3,*]

[1] State Key Laboratory of Swine and Poultry Breeding Industry, College of Animal Science, South China Agricultural University, Guangzhou 510642, China; h2473552637@stu.scau.edu.cn (Z.H.); 15521277019@163.com (M.L.); 15360428153@163.com (Z.H.); yanxiliang1991@163.com (X.Y.); wuyinbao@scau.edu.cn (Y.W.)

[2] Guangdong Provincial Key Lab of Agro-Animal Genomics and Molecular Breeding, South China Agricultural University, Guangzhou 510642, China

[3] National Engineering Research Center for Breeding Swine Industry, South China Agricultural University, Guangzhou 510642, China

[4] College of Life Science, South China Agricultural University, Guangzhou 510642, China; mpeiqi-ang@scau.edu.cn (P.M.); jiangjun@scau.edu.cn (J.J.)

[5] Institute of Quality Standard and Monitoring Technology for Agro-Products of Guangdong Academy of Agricultural Sciences, Guangzhou 510642, China; wangxuguangzhou@126.com

* Correspondence: ywang@scau.edu.cn; Tel.: +86-20-85280279; Fax: +86-20-85280740

Captions:

Table S1. The growth performance of British Shorthair cats.

Table S2. Abundance of total bacterial counts in fresh feces of different groups of British shorthair cats.

Table S3. The chemical composition of RE and RE100.

Figure S1. Schematic structure of the respiratory metabolism chamber.

Figure S2. Differential analysis of phylum-level bacteria between the two groups.

Figure S3. Differential analysis of genus-level bacteria between the two groups.

Figure S4. A typical gas chromatogram of the chemical constituents of hexane extract.

Table S1. The growth performance of British Shorthair cats.

Indexes	CK	RE	RE100
initial weight （kg）	4.92±0.52^{a}	4.03±0.29^{a}	4.39±0.14^{a}
final weights （kg）	5.04±0.47^{a}	4.11±0.28^{a}	4.50±0.11^{a}
ADFI（g/d）	78.41±1.37^{a}	79.91±0.43^{a}	71.85±3.33^{a}
ADG（g/d）	4.29±1.79^{a}	3.10±2.00^{a}	4.05±1.56^{a}

Values are expressed as mean ± SE. Different superscripts letters (a, b, c) within the same row indicate statistically significant differences.

Table S2. Abundance of total bacterial counts in fresh feces of different groups of British shorthair cats.

Group	Log (copies/g)
CK	9.02±0.51^{a}
RE	9.82±0.57^{a}
RE100	9.72±0.31^{a}

Values are expressed as mean ± SE. Different superscripts letters (a, b, c) within the same row indicate statistically significant differences.

Table S3. The chemical composition of RE and RE100.

	Compound	Percentage (%)	
		RE	RE100
lipids	9-Octadecenamide, (Z)-	6.93%	3.82%
	Aminocaproic acid	1.98%	2.35%
	Cholest-5-en-3-ol (3-, acetate	1.00%	1.04%
	Pregnan-20-one, 3-(acetyloxy)-5,6-epoxy-6-methyl-, cyclic 20-(1,2-ethanediyl acetal), (356-	0.82%	0.93%
	15-OxoEDE	0.54%	0.31%
	3,914,15-Diepoxypregn-16-en-20-one, 3,1118-triacetoxy-	0.51%	0.24%
	1-Stearoyl-2-linoleoyl-sn-glycero-3-phospho-(1'-rac-glycerol)	0.47%	0.13%
	Prost-13-en-1-oic acid, 9-(methoxyimino)-11,15-bis[(trimethylsilyl)oxy]-, trimethylsilyl ester, (8.xi.,12.xi.)-	0.30%	0.23%
	Octadecanoic acid, 1-[(tetradecyloxy)carbonyl]pentadecyl ester	0.30%	0.30%
	15-Deoxy-.DELTA.12,14-prostaglandin J2-2-glycerol ester	0.26%	0.27%
	1'-Carboethoxy-1'-cyano-12dihydro-17propionoxy-3'H-cycloprop[1,2]androsta-1,4,6-trien-3-one	0.19%	0.09%
	4-Androsten-9fluoro-17methyl-361117tetra-ol, tetra-trimethylsilyl	0.18%	0.23%
	1,2-Di-(9Z-octadecenoyl)-sn-glycero-3-phospho-(1'-myo-inositol)	0.17%	0.04%
	19,20-DiHDPA	0.10%	0.10%
	1,2-Dioleoyl-sn-glycero-3-phosphoethanolamine-N-methyl	0.09%	0.03%
	21-hydroxypregnenolone 2	0.07%	0.00%

	1-Hexadecyl-2-(9Z-octadecenoyl)-sn-glycero-3-phosphoethanolamine	0.07%	0.01%
	1-Octadecanoyl-2-(5Z,8Z,11Z,14Z-eicosatetraenoyl)-sn-glycero-3-phosphoethanolamine	0.05%	0.11%
	Prosta-8(12),13-dien-1-oic acid, 9-(methoxyimino)-15-[(trimethylsilyl)oxy]-, trimethylsilyl ester	0.04%	0.05%
Flavonoids	naringenin 2	0.24%	0.28%
	Myricitrin	9.01%	9.26%
	2-Hydroxychalcone	0.71%	0.21%
	Hesperitin 1	0.22%	0.07%
	2'-Hydroxy-2,4,4',6'-tetramethoxychalcone	0.21%	0.48%
	Flavanone diacetylhydrazone	0.15%	0.08%
	(E)-1-(2-Hydroxy-4,6-dimethoxyphenyl)-3-phenylprop-2-en-1-one	0.13%	0.01%
	4-Hydroxy-2',4',6'-trimethoxychalcone	0.08%	0.04%
	2',4,6'-Trimethoxychalcone	0.06%	0.00%
	4'-Hydroxychalcone	0.05%	0.07%
	2'-Hydroxy-3,4,5-trimethoxychalcone	0.04%	0.00%
	7-Hydroxy-3-(4-methoxyphenyl)-4-phenylcoumarin	0.04%	0.04%
organic acids	Triphenyl phosphate	2.40%	2.20%
	Phthalic acid, (2-chlorocyclohexyl)methyl isobutyl ester	1.69%	1.46%
	Phthalic acid, butyl hept-3-yl ester	0.51%	0.63%
	Spiro[9,9']difluorene-2,2'-dicarboxylic acid, 7,7'-dinitro-	0.43%	0.30%
	Glafenin	0.14%	0.07%
	Acetic acid, (2-propenylthio)-	0.07%	0.06%
hydrocarbons	Heptadecane	0.81%	0.90%
	Decane, 5-ethyl-5-methyl-	0.64%	0.89%
	Triacontane, 11,20-didecyl-	0.63%	0.53%
	2,6-Dimethyldecane	0.61%	0.89%
	Undecane, 2-methyl-	0.51%	0.52%
	Undecane, 4,7-dimethyl-	0.41%	0.60%
	Tridecane	0.40%	0.49%
	Octadecane, 3-ethyl-5-(2-ethylbutyl)-	0.27%	0.29%
	Decane, 2,4,6-trimethyl-	0.26%	0.29%
	Dibenz[a,h]anthracene, 5,6,12,13-tetrahydro-	0.05%	0.19%
	Fluorene	0.04%	0.05%
	[DAla2] Dynorphin A (1-9), porcine	1.06%	0.74%
	L-Tyrosine	0.58%	0.68%
	Tazobactam	0.56%	0.22%
	Conotoxin MI	0.46%	0.87%
amino acids and their derivatives	GTP-Binding Protein Fragment, Go alpha	0.30%	0.13%
	Hippuric acid, methyl ester	0.19%	0.00%
	Dynorphin A (3-13), porcine	0.12%	0.12%
	Conotoxin SI	0.12%	0.19%
	(Glp1)-Apelin-13, bovine, human	0.09%	0.05%
	Conotoxin IMI	0.09%	0.00%
	Conotoxin GI	0.06%	0.05%

	L-Canavanine	0.03%	0.01%
ligans	7,8-Dimethoxycoumarin	1.42%	1.04%
and cou-	Podofilox	0.40%	0.00%
marins	Rutamarin	0.35%	0.12%
	Tetrandrine	0.25%	0.09%
	7-Hydroxy-4-(methoxymethyl)coumarin	0.21%	0.80%
terpe- noids	2,4-Epoxymenthanophenanthren-12-one,1,2,3,4,4a,4b,5,6,7,8,10,10a-dodecahydro-1-hydroxy-1-(2-ethoxyvinyl-2-propyl-4b-methyl-7,7-(2,5-dioxocyclopentyl)-	0.41%	0.36%
	12-O-Acetylingol 8-tiglate	0.40%	0.43%
	Rhodopin	0.16%	0.00%
	9-Desoxo-9-x-acetoxy-3-desoxy-7.8.12-tri-O-acetylingol-3-one	0.14%	0.03%
	9-Desoxy-9x-chloroingol 3,7,8,12-tetraacetate	0.14%	0.10%
	Lycopene	0.12%	0.00%
	9-Desoxo-9-x-acetoxy-3,8,12-tri-O-acetylingol	0.12%	0.15%
	.psi.,.psi.-Carotene, 3,4-didehydro-1,2,7',8'-tetrahydro-1-methoxy-2-oxo-	0.12%	0.06%
	5Pregn-16-en-20-one, 312dihydroxy-, diacetate	0.12%	0.03%
	(2,4a,9,9a-Tetrakis(acetyloxy)-2,7b-dihydroxy-1,1,6,8-tetramethyl-5-oxo-1b,3,4,5,7a,7b,8,9-octahydro-1H-cyclopropa[3,4]benzo[1,2-E]azulen-3(1ah,2H)-yl)methyl acetate	0.11%	0.01%
	Megestrol acetate	0.11%	0.03%
	.psi.,.psi.-Carotene, 3,4-didehydro-1,1',2,2'-tetrahydro-1'-hydroxy-1-methoxy-	0.10%	0.07%
	Pregna-5,8-diene-311diol-20-one diacetate	0.10%	0.09%
	Phorbol 12,13-Dibutyrate	0.09%	0.11%
	3,8,12-Tri-O-acetoxy-7-desoxyingol-7-one	0.07%	0.05%
	11-Hydroxy-.DELTA.-9-tetrahydrocannabinol, bis(trimethylsilyl) ether	0.07%	0.10%
	Pregnan-20-one, 3-(acetyloxy)-5,6-epoxy-, cyclic 20-(1,2-ethanediyl acetal), (356-	0.05%	0.00%
	Cholestan-3-one, cyclic 1,2-ethanediyl aetal, (5-	0.02%	0.00%
alka-	Norcodeine, N-trimethylsilyl-, trimethylsilyl ether	0.63%	0.33%
loids	Aconitine	0.54%	0.06%
	1-(3-Fluoro-phenyl)-2,6,6-trimethyl-3-(2,2,2-trichloro-ethyl)-1,5,6,7-tetrahydro-indol-4-one	0.23%	0.01%
	indole-3-acetamide 4	0.19%	0.08%
	Homatropin	0.07%	0.00%
	3-(Methylaminopentyl)-5-(4-bromophenyl)-1-(4-nitrophenyl)-1H-pyrazole	0.06%	0.10%
	1H-[1,2]-Dithiolo[3,4-c]quinoline-1-thione, 4,5-dihydro-5-(2-furoyl)-4,4-dimethyl-	0.05%	0.03%
	3,4-Dihydroisoquinolin-7-ol, 1-[4-hydroxybenzyl]-6-methoxy-	0.05%	0.00%
	Thieno[2,3-c]furan-3-carbonitrile, 2-amino-4,6-dihydro-4,4,6,6-tetramethyl-	0.03%	0.01%
	3-Hydroxybromoazepam, bis(trimethylsilyl)- deriv	0.02%	0.01%
Pphe-	piceatannol 1	0.40%	0.34%
nolic ac-	2,6-Di-tert-butyl-4-methoxyphenol	0.27%	0.98%
ids	4-hydroxyphenylacetic acid	0.18%	0.15%
	2,4,6-Trihydroxybenzophenone	0.10%	0.20%
nucleo-	2-Chloroadenosine	0.21%	0.23%
tides	Cytidine	0.19%	0.10%
and their	2'-Deoxycytidine 5'-triphosphate	0.12%	0.04%
	Thymidine 5'-monophosphate	0.07%	0.01%

deriva-tives			
others	N-Desmethylverapamil	33.75%	39.48%
	Cyclohexasiloxane, dodecamethyl-	2.99%	1.74%
	3-Isopropoxy-1,1,1,7,7,7-hexamethyl-3,5,5-tris(trimethylsiloxy)tetrasiloxane	1.45%	1.68%
	Borane, diethyl[1-ethyl-2-(trimethylstannyl)-1-propenyl]-	1.38%	0.48%
	Cyclopentasiloxane, decamethyl-	1.34%	0.75%
	Cyclooctasiloxane, hexadecamethyl-	1.29%	3.93%
	Isoproturon	1.02%	0.32%
	1-[2,4-Bis(trimethylsiloxy)phenyl]-2-[(4-trimethylsiloxy)phenyl]propan-1-one	0.77%	0.40%
	Cyclotrisiloxane, hexamethyl-	0.75%	0.45%
	Cyclononasiloxane, octadecamethyl-	0.66%	4.05%
	Quinomethionate	0.60%	0.23%
	2-Chloro-3,6-diphenyl-3,4-dihydro-2H-1,3,2-oxazaphosphinine 2-oxide #	0.50%	0.58%
	1-Iodoundecane	0.46%	0.38%
	5-(p-Aminophenyl)-4-(O-tolyl)-2-thiazolamine	0.45%	0.69%
	Thymolphthalein	0.42%	0.02%
	Felodipine	0.40%	0.48%
	Cyclotetrasiloxane, octamethyl-	0.40%	0.02%
	lactose 1	0.34%	0.31%
	7-Chloro-3-[3,4-dichlorophenyl]-1-[[3-[dimethylamino]propyl]imino]-1,3,4,10-tetrahydro-9(2H)-acridinone	0.33%	0.29%
	1,4:5,8-Dimethanonaphthalene-2,3-diol, 5,6,7,8,9,9-hexachloro-1,2,3,4,4a,5,8,8a-octahydro-, diacetate, (12344a588a-	0.26%	0.24%
	Silane, diethyl(2-isopropylphenoxy)nonyloxy-	0.26%	0.00%
	Phenyl 4-[(trimethylsilyl)amino]-2-[(trimethylsilyl)oxy]benzoate	0.26%	0.07%
	Tricyclo[4.2.1.0(2,5)]non-7-ene, 3,4-di(tris(trimethylsilyloxy)silyl)-	0.23%	0.35%
	Benzoic acid, 4-methyl-2-trimethylsilyloxy-, trimethylsilyl ester	0.23%	0.24%
	Pentamethylcyclopentadienyl-trichlorogermyl-ethylisonitril-carbonyl-trimethylphosphan-tungsten	0.23%	0.32%
	5-Endo-hydroxy-protoadamantane	0.22%	0.49%
	Methiocarb sulfoxide	0.20%	0.08%
	2,4-Imidazolidinedione,5-[3,4-bis[(trimethylsilyl)oxy]phenyl]-3-methyl-5-phenyl-1-(trimethylsilyl)-	0.18%	0.00%
	D-Glucopyranoside, methyl 2,3,4,6-tetra-O-methyl-	0.17%	0.32%
	Allylchlorodimethylsilane	0.15%	0.44%
	Thallium O,O'-bis(4-chlorophenyl)dithiophosphate	0.15%	0.09%
	Haloxazolam	0.14%	0.08%
	Triprolidine	0.12%	0.01%
	14H-Dibenzo[a,j]xanthene, 14-methyl-	0.11%	0.13%
	Naphtho[2,3-c]furan-1(3H)-one,3a,4,9,9a-tetrahydro-6-hydroxy-4-(4-hydroxy-3-methoxy-phenyl)-7-methoxy-, [3aR-(3a49a]-	0.10%	0.02%
	[1]Benzopyrano[4,3-b]indole, 6,11-dihydro-	0.08%	0.00%

2-Amino-3-cyano-4,6-bis(4-cyanophenyl)-4-methyl-cyclohexa-1,5-dien-1,3-dicarboxylic acid, diethyl ester	0.08%	0.00%
2,6-Bis(4-azidobenzylidene)-4-methylcyclohexanone	0.07%	0.01%
Benzothiophene-2-carboxylic acid, 4,5-dihydro-3-acetamido-7-chloro-6-cyano-, ethyl ester	0.07%	0.04%
Bendazol	0.06%	0.03%
Acetyl bromide	0.05%	0.00%
Sulfameter	0.05%	0.00%
{Methanediylbis[(3,4,6-trichlorobenzene-2,1-diyl)oxy]}bis(trimethylsilane)	0.05%	0.00%
5,12d-Ethano(furo[2,3,4-mn]oxepino[2,3,4-ed]anthracen-9-ol-2-one),6-methyl-12acetoxy-2a,3,4,4a,5,7,8a-octahydro-	0.05%	0.10%
1H-Cyclopent[c]isoxazole,1-[2,3:5,6-bis-O-(1-methylethylidene)-d-mannofuranosyl]hexa-hydro-4,5,6-tris(phenylmethoxy)-, [3aR -(3a45	0.04%	0.00%
Methanol, [4-(1,1-dimethylethyl)phenoxy]-, acetate	0.03%	0.01%
4,7-Methano-1H-indene, 4,5,6,7,8,8-hexachloro-3a,4,7,7a-tetrahydro-	0.02%	0.02%
total	100%	100%

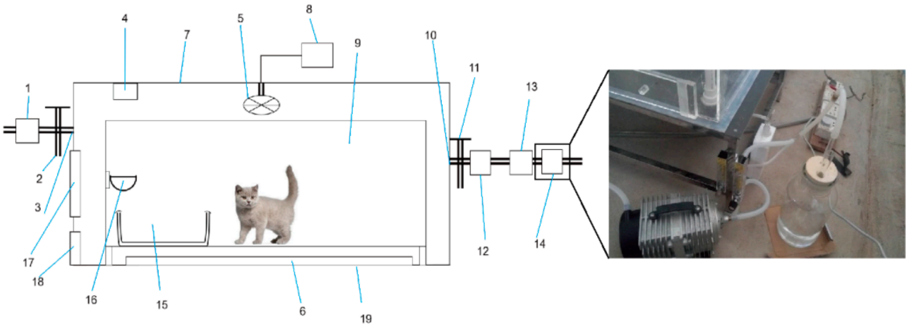

Figure S1. Schematic structure of the respiratory metabolism chamber. (1) air filter. (2) air inlet valve. (3) air inlet. (4) temperature sensor. (5) fan. (6) gasket. (7) outer plexiglass cover. (8) power supply. (9) animal metabolism cage. (10) air outlet. (11) air outlet valve. (12) flow meter. (13) gas pump. (14) air extraction system. (15) litter bowl. (16) suspended cat food bowl, water bowl. (17) feeder and litter cleaning baffle. (18) freely removable baffle. (19) bottom plate.

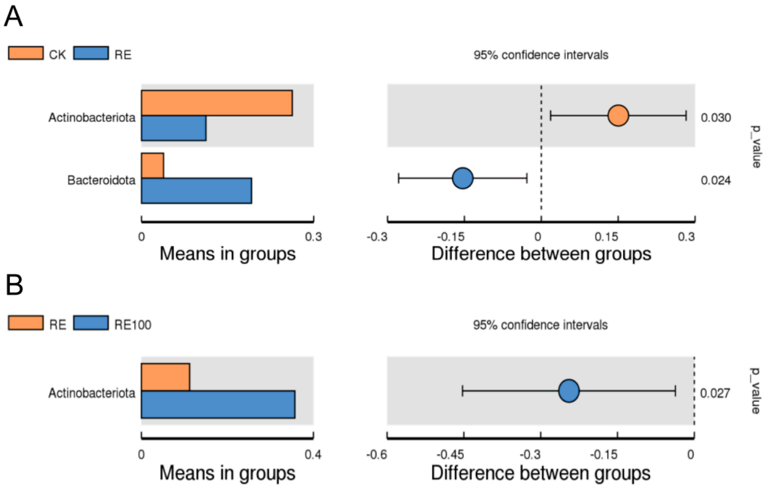

Figure S2. Differential analysis of phylum-level bacteria between the two groups. (A) the CK group and RE group. (B) The CK group and RE100 group.

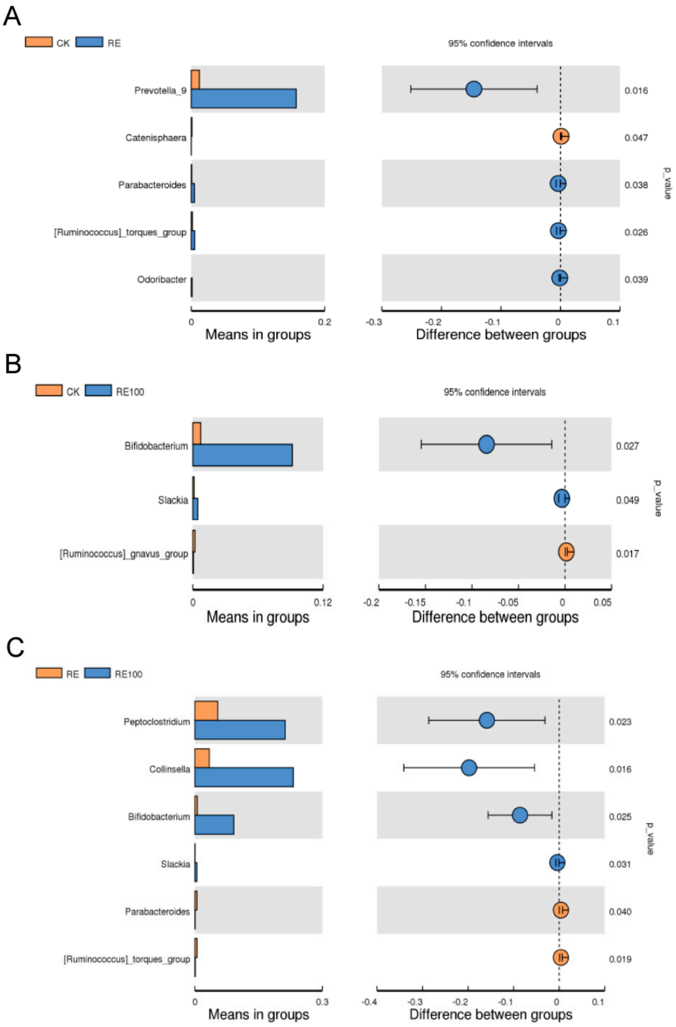

Figure S3. Differential analysis of genus-level bacteria between the two groups. (A) The CK group and RE group. (B) The CK group and RE100 group. (C) The RE group and RE100 group.

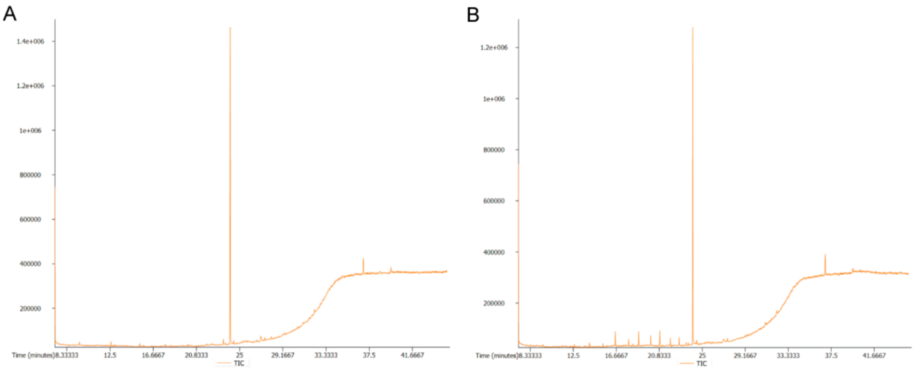

Figure S4. A typical gas chromatogram of the chemical constituents of hexane extract. (A) Typical gas chromatogram of the chemical constituents of rosemary extract. (B) Typical gas chromatogram of the chemical composition of the less than 100 Da fraction of rosemary extract.
